# Resistant/Refractory Hypertension and Sleep Apnoea: Current Knowledge and Future Challenges

**DOI:** 10.3390/jcm8111872

**Published:** 2019-11-05

**Authors:** Grace Oscullo, Gerard Torres, Francisco Campos-Rodriguez, Tomás Posadas, Angela Reina-González, Esther Sapiña-Beltrán, Ferrán Barbé, Miguel Angel Martinez-Garcia

**Affiliations:** 1 Pneumology Department, La Fe University and Polytechnic Hospital, 46026 Valencia, Spain; graceoscullo@gmail.com (G.O.); t.posadas21@gmail.com (T.P.); 2 Group of Translational Research in Respiratory Medicine, IRBLleida, Hospital Universitari Arnau de Vilanova and Santa Maria, 25198 Lleida, Spain; gtorres@gss.scs.es (G.T.); esther.sb91@gmail.com (E.S.-B.); febarbe.lleida.ics@gencat.cat (F.B.); 3 Respiratory Department, Hospital Valme, 41014 Seville, Spain; fracamrod@gmail.com (F.C.-R.); angelareinagonzalez@separ.es (A.R.-G.); 4 Institute of Biomedicine of Seville (IBiS), 41013 Seville, Spain; 5 Center of Biomedic Research in Respiratory Diseases (CIBERES), 28029 Madrid, Spain

**Keywords:** Resistant hypertension, Refractory hypertension, Obstructive sleep apnoea, Continuous positive airway pressure

## Abstract

Hypertension is one of the most frequent cardiovascular risk factors. The population of hypertensive patients includes some phenotypes whose blood pressure levels are particularly difficult to control, thus putting them at greater cardiovascular risk. This is especially true of so-called resistant hypertension (RH) and refractory hypertension (RfH). Recent findings suggest that the former may be due to an alteration in the renin–angiotensin–aldosterone axis, while the latter seems to be more closely related to sympathetic hyper-activation. Both these pathophysiological mechanisms are also activated in patients with obstructive sleep apnoea (OSA). It is not surprising, therefore, that the prevalence of OSA in RH and RfH patients is very high (as reflected in several studies) and that treatment with continuous positive airway pressure (CPAP) manages to reduce blood pressure levels in a clinically significant way in both these groups of hypertensive patients. It is therefore necessary to incorporate into the multidimensional treatment of patients with RH and RfH (changes in lifestyle, control of obesity and drug treatment) a study of the possible existence of OSA, as this is a potentially treatable disease. There are many questions that remain to be answered, especially regarding the ideal combination of treatment in patients with RH/RfH and OSA (drugs, renal denervation, CPAP treatment) and patients’ varying response to CPAP treatment.

## 1. Introduction

Hypertension is one of the most common modifiable risk factors for the development of cardiovascular disease and mortality. The global prevalence of hypertension, based on office blood pressure (BP) measurements, has been estimated at 1.13 billion individuals worldwide in 2015, with a prevalence in adults of 30–45% [1], and with half of these patients not achieving any adequate BP control [2]. There are some difficult-to-treat phenotypes, such as those patients with resistant hypertension (RH) [3] and refractory hypertension (RfH) [4]. 

RH and RfH are characterized by an incomplete response to antihypertensive therapy, which makes it particularly challenging to identify potentially modifiable associated factors that could help to reduce BP levels. This identification is essential, however, in the light of the high cardiovascular impact of RH and RfH, as they are associated with a 50% higher probability of developing a cardiovascular event or damage to target organs than controlled hypertensive patients [5] 

Obstructive sleep apnoea (OSA) is a common sleep disorder characterised by recurrent episodes of upper airway obstruction that provoke intrathoracic pressure changes, sleep fragmentation, intermittent hypoxia and increased sympathetic activity, triggering a variety of intermediate mechanisms that mediate various clinical outcomes, including hypertension and a propensity to a non-dipper circadian BP pattern [6]. Recent studies report a prevalence of moderate-to-severe OSA, defined as an apnoea–hypopnoea index (AHI) of 15 or more events/h, in 10% to 21% of the general population, and up to 49% in elderly subjects [7,8,9,10]. The prevalence of moderate-to-severe OSA worldwide has been estimated at 425 million in adults aged from 30 to 69 years [11].

OSA and hypertension are closely related. OSA triggers direct and intermediate mechanisms associated with the development and maintenance of hypertension, such as sympathetic hyperactivity [6]. The prevalence of OSA is as high as 30–50% in hypertensive patients [12], increasing to 70–85% in patients with RH [13,14] and peaking at more than 90% in those with RfH [15]. In fact, of all the various secondary causes of RH, OSA has been identified as the most common disorder associated with RH, accounting for more than half of the patients in some studies [16]. Furthermore, it has been demonstrated that OSA treatment with continuous positive airway pressure (CPAP) reduces BP by around 2–2.5 mmHg in patients with hypertension and by up to 5 mmHg in those with RH [17]. All this evidence suggests that OSA and its treatment may play an important role in RH. 

In this review, we will cover the different aspects of the relationship between RH and OSA, including the epidemiology of RH and RfH, the clinical association with OSA, the pathophysiological links between the two disorders and the different therapies for RH and RfH (with a special focus on OSA treatment), as well as potential future challenges in the interactions between OSA and RH. 

## 2. Resistant and Refractory Hypertension Phenotypes: Definition and Epidemiology

RH was defined in the first American Heart Association (AHA) statement as those forms of hypertension with no identifiable cause in which BP levels remain uncontrolled despite the use of at least three antihypertensive drugs (including a diuretic) prescribed at optimal doses or are controlled only by treatment with four or more antihypertensive drugs [18]. Based on this statement, the prevalence of RH ranges from 12% to 15%, depending on whether the latter group of patients is taken into consideration [19,20,21].

A more recent statement that took into account new evidence reported in the latest clinical studies adapted the thresholds of clinical BP and provided greater details of the type, dose and frequency of the drugs that patients should usually be taking to fulfil the definition of RH. This statement also points out the importance of performing 24 h ambulatory blood pressure monitoring (ABPM) in the assessment process for this type of patient [3]. 

One of the most important contributions in recent years has been the definition of a particular subgroup within RH patients. Acelajado et al. [22], after a retrospective study of patients with RH undertaken by a specialist hypertension unit, proposed a specific definition for those subjects who never achieved BP control, regardless of the number of drugs added or the drug regime implemented: this type of patient should be considered to be suffering from refractory hypertension (RfH). The patients who satisfied this definition were taking an average of six drugs.

However, the first definition of RfH proposed by Acelajado et al. [22] has been modified to include the requirement of antihypertensive treatment with a strict drug regime that controls the volume overload. Accordingly, the AHA’s latest statement on hypertension [3] defined RfH as those forms of hypertension that remain uncontrolled despite the administration of at least five antihypertensive drugs, including a long-acting thiazide-like diuretic and a mineral-corticoid receptor antagonist. Based on this definition, the prevalence of this hypertension phenotype is about 3% of patients with RH [23].

Therefore, the key point for the fulfilment of the definition of RfH that satisfies all these requirements is the failure to achieve BP control, despite adding more drugs and maximising the most effective drugs while also reducing the volume overload.

Consequently, some significant differences have been reported in the clinical profiles of subjects with RH and those with RfH. RfH patients seem to be younger [24,25,26], with a higher prevalence of diabetes [24,25], cardiovascular disease [23,24], stroke [25] and chronic kidney disease [24,25].

## 3. Clinical and Epidemiological Association with OSA

High-quality evidence has shown that OSA and hypertension are closely related. It has been established that about 50% of OSA patients suffer from hypertension and that roughly 30–50% of hypertensive patients have OSA [12,27,28]. Several clinic-based studies (and a few epidemiological studies) have shown that the association between OSA and RH is even stronger.

Cross-sectional studies have reported a high prevalence of OSA among patients with RH, ranging from 60–90%, depending on the AHI threshold used to define OSA [13,14,16,29,30,31,32,33,34] (Table 1). The relationship between these two disorders was first described in 2001 by Logan et al. in a small series of 24 men and 17 women with RH who presented a surprisingly high prevalence (83%) of OSA [13]. Other researchers subsequently confirmed the close relationship between both disorders. Pedrosa et al. closely studied a cohort of 125 RH patients for secondary causes of RH and found that moderate-to-severe (AHI ≥ 15 events/h) OSA was by far the most common condition associated with RH, accounting for 64% of all causes, with the second most common cause, primary aldosteronism, a long way behind at 5.6% [16]. In a similar study of 204 patients, OSA of any severity (AHI ≥ 5 events/h) was the most prevalent disorder associated with RH, in 72.1% of cases [32]. 

Other studies have shown that the prevalence of OSA is significantly increased in patients with RH compared to those with controlled hypertension. In a case control study of 63 patients with RT and 63 with controlled hypertension, OSA was present in 71% of the patients in the first group, compared to only 38% in the second (*p* < 0.001) [35]. Ruttanaumpawan et al. also found a greater prevalence of OSA in RH compared to controlled hypertension (81 vs. 55%, *p* = 0.03) [14]. In both studies, OSA was a strong independent predictor of RH, with an adjusted OR of 3.99 to 4.8. In a large retrospective cohort study using a database composed of 470,386 hypertensive individuals whose OSA diagnosis was retrieved by means of ICD-9 codes, OSA was present in 9.6% of patients with RH, compared to 6.8% in non-RH patients [36]. 

It is well known that there are significant racial and ethnic disparities in hypertension prevalence, with higher rates of RH in non-Hispanic blacks. In this respect, two studies have investigated the association between OSA and RH in the black population [31,37]. The Jackson Heart Sleep Study analysed 664 black adults with hypertension by means of home respiratory polygraphy [37]. After adjustment for confounders, patients with moderate-to-severe OSA (AHI ≥ 15 events/h) had 2.0 times (95% CI 1.14–3.67) higher odds of having RH than those with mild OSA, or no OSA. Another study conducted on 200 black participants with hypertension in a primary care setting showed that patients with RH were nearly 2.5 times more likely to be at high OSA risk than those with controlled hypertension, after adjusting for age, gender and medical co-morbidities [31]. In this study, however, OSA risk was assessed by using a screening tool and not a sleep test.

Abdel-Kader et al. have addressed the association between OSA, RH and chronic kidney disease in a cohort composed of 224 community-based subjects without chronic kidney disease, 88 patients with non-dialysis-dependent chronic kidney disease and 95 with end-stage renal disease by means of unattended home polysomnography and home BP measurements [38]. The authors observed a significant association between severe OSA (AHI ≥ 30 events/h) and end-stage renal disease (OR 7.1, 95% CI 2.2–23.2), whereas patients with non-dialysis-dependent chronic kidney disease and severe OSA did not appear to be at increased risk of RH (OR 1.2, 95% CI 0.4–3.7) after accounting for confounders such as age, sex, race and body mass index.

It is notable that OSA patients with RH do not usually present typical OSA symptoms, particularly excessive daytime somnolence (EDS). In this respect, one study observed that only 70% of patients with OSA and RH were snorers and very few had witnessed apnoeas [29], whereas another study showed that the Epworth Sleepiness Score (ESS) was not an adequate screening tool for OSA in patients with RH, since less than 45% had EDS and the sensitivity and specificity of the ESS were below 55% [39]. According to these findings, OSA screening questionnaires based on symptoms would serve for little in patients with RH. However, various studies that have assessed OSA risk via screening questionnaires (usually the Berlin Questionnaire) have observed that two thirds of those subjects attending an RH clinic were identified as being at high risk of OSA, and that the risk of OSA, as assessed by this screening test, was significantly higher than in patients with controlled hypertension [31,39]. 

All the aforementioned evidence has led the scientific community to consider OSA as an important contributing factor to hypertension and RH. In 2003, the *Seventh Report of the Joint National Committee on Prevention, Detection, Evaluation and Treatment of High Blood Pressure* included OSA as a common identifiable cause of hypertension [40], and in the 2017 hypertension guidelines, OSA was acknowledged as a secondary contributor to RH and OSA screening was recommended for patients with RH or RfH [41]. Moreover, considering that most of these patients are non-sleepy and may not have typical OSA symptoms, it is recommended that every patient with at least chronic snoring and RH should undergo a sleep study, due to the high risk of OSA.

Although it is unclear whether RfH is simply the most severe extreme of the RH spectrum or whether it really constitutes a different clinical phenotype, it undoubtedly carries the greatest cardiovascular risk. So far, however, only one study has investigated the association between OSA and RfH [15]. In this multicentre cross-sectional Spanish study, Martinez-Garcia et al. analysed 229 consecutive patients with RH diagnosed by means of 24 h ABPM. All the participants underwent a home respiratory polygraphy. Compared with RH patients, those with RfH had a twofold greater risk of having severe OSA (OR 1.9, 95% CI 1.02–3.8). The prevalence of moderate (AHI ≥ 15 events/h) and severe (AHI ≥ 30 events/h) OSA was 95.2% and 64.3%, respectively, in the RfH group, compared to 81.8% and 48.6% for the RH group (*p* < 0.05 for both comparisons). 

## 4. Pathophysiological Links with Obstructive Sleep Apnoea (OSA)

**Figure 1:**
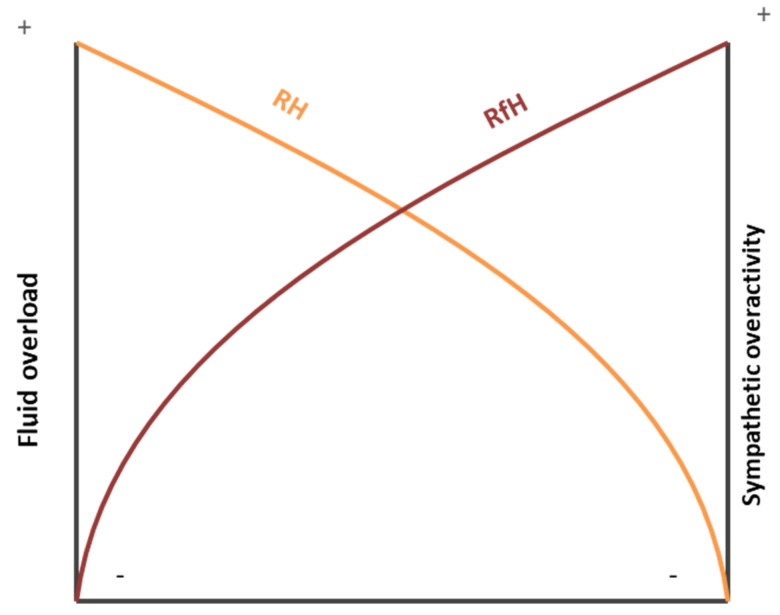
Pathophysiology of resistant and refractory hypertension. RH: resistant hypertension; Rfh: refractory hypertension.

As there appear to be crucial differences between the pathophysiology of RH and RfH (Figure 1), there may also be significant differences in the ways that these two conditions are related to OSA.

### 4.1. Resistant Hypertension

Despite the different pathophysiological mechanisms behind RH, fluid overload is considered to be the main one [42]. Sympathetic hyperactivity also makes a contribution [43]. An excess of fluid retention is caused by excessive salt intake and salt sensitivity [44], age, obesity, chronic kidney disease and, most particularly, hyperdosteronism [45], which is associated with overactivation of the renin–angiotensyn aldosterone axis. A wide range of studies have demonstrated the key role played by intensive diuretic treatment, including a mineralocorticoid receptor antagonist, in achieving BP control of RH patients, and this is considered to demonstrate the transcendental importance of this mechanism in RH [46,47,48].

The high prevalence of OSA in resistant hypertensive patients could be explained by fluid retention, mainly as a result of hyperaldosteronism. The negative thoracic pressure associated with sleep apnoea causes fluid to move from the legs to the neck area when the patient is lying down [49], resulting in an oedema of the parapharyngeal tissues that could propitiate upper airway obstruction. The more severe the OSA, the greater the related sympathetic activity, and the subsequent stimulation of the renin-angiotensin-aldosterone axis. This increased fluid retention can therefore imply not only higher BP but also more parapharyngeal oedemas and apnoeas, thus creating a fatal vicious circle.

### 4.2. Refractory Hypertension

As mentioned above, RfH is defined as BP that remains uncontrolled despite therapy with at least five drugs, including an intensive diuretic treatment that maximizes volume depletion, a long-acting thiazide-like diuretic and a mineral-corticoid receptor antagonist [3]. It is not surprising, therefore, that sympathetic over-activity is believed to be the main mechanism behind RfH, as confirmed by the results of a comparative clinical study by Dudenbostel et al. of RfH and RH patients [23]. They found a lower fluid content in those patients with RfH, reflected in a lower sodium dietary intake and lower thoracic fluid content, assessed via an impedance cardiography study. They also reported findings in clinical and biomarker measures that suggested greater sympathetic activity, such as higher office resting and ambulatory heart rate and higher 24 h urinary normetanephrine levels.

As in the case of RfH, sympathetic over-activity in OSA patients seems to be the main pathophysiological mechanism explaining the syndrome’s pressor effect [6,15,50]. In these patients, respiratory events and their related intermittent hypoxia trigger sympathetic hyperactivation, leading to a high incidence and prevalence of hypertension. Other less important mechanisms, such as oxidative stress, inflammation and endothelial dysfunction, also contribute to this effect [51]. For all these reasons, it seems clear that this common pathophysiological syndrome has significant clinical implications.

## 5. Multidimensional Treatment

Despite the significant progress made in recent years with respect to the treatment of RH, several aspects remain unclear. The situation is even worse as regards RfH, as there is nothing to test beyond hypothesis [52].

### 5.1. Lifestyle Changes and Diet

The same recommendations about lifestyle changes given to the general hypertensive population could also be considered suitable for RH patients, and so the need for weight reduction, sodium restriction (2 gr/day), moderation of alcohol consumption and regular physical exercise (3–5 sessions of 30–40 min weekly) should be advocated.

Nevertheless, some specific points still need to be clarified. Although weight loss does have a significant effect on BP reduction, and might improve OSA when it is present [53], no trials to date have demonstrated how effective it is in reducing BP specifically in RH patients. Furthermore, there are doubts regarding salt intake. There is a significant linear relationship between sodium restriction and BP reduction in subjects with RH, even at low levels (1.5–3 gr/day), and restrictions on salt intake could reduce the severity of any concomitant OSA [54]. Although the clinical studies on this topic [44,55] have confirmed reductions in daytime and night time BP, they have included only small numbers of patients and have not cleared any doubts about long-term efficacy. Moreover, there seems to be a U-shaped relationship between sodium restriction and cardiovascular mortality, as a radical restriction could increase cardiovascular risk, despite lowering BP [56]. Other dietary measures that have proved effective in reducing BP in the general hypertensive population, such as DASH (dietary approach to stop hypertension) and the Mediterranean diet [57,58,59], have still not been specifically assessed in RH patients.

### 5.2. Renal Denervation

The initial studies testing renal denervation treatment for RH patients raised expectations for this procedure as a possible definitive therapy for both RH and RfH [60,61]. Furthermore, the efficacy of this procedure did not seem to be influenced by the presence of OSA or otherwise, or its treatment [62,63]. Moreover, it might result in additional benefits for OSA and decrease its severity when it is concomitant [62]. However, the SYMPLICITY III study (NTC02041130) on 535 patients randomized to renal denervation vs. sham procedure found no significant benefits for systolic blood pressure and raised doubts about efficacy of the procedure, the most appropriate techniques for performing it and the most suitable candidates [64]. Therefore, as stated in most of the current guidelines, it is necessary to undertake more research to clarify the indications for this procedure in RH patients [1,3]. 

### 5.3. Pharmacological Treatment

Pharmacological therapy has constituted the primary approach to the treatment of RH to date and it has provided the main advances made in recent years, although some RfH patients never achieve BP control. There is no longer any doubt about the efficacy of spironolactone in improving BP control and it should be added to the baseline treatment, if possible, as a fourth drug (this treatment should already include a diuretic at optimal dose) [48]. Spironolactone might also significantly reduce the severity of OSA, when it is present [65,66], and this could also be true of other diuretics [67,68]. However, the use of spironolactone is limited by side effects such as gynaecomastia, reported in 5% of patients taking it [69], and contraindications such as chronic kidney disease (a glomerular filtration rate under 45 mL/min and potassium level above 4.5 mmol/L), because it significantly increases the risk of hyperkalemia, particularly in a dual renin angiotensin aldosterone system (RAAS) blockade regimen [70]. Two further points need to be taken into account. On the one hand, the results of the REACH registry, which included 6790 patients meeting the criteria of RH, followed for a mean time of four years, showed that, even with good BP control, a higher number of anti-hypertensive drugs may not reduce the long-term risk of an adverse cardiovascular outcome [19]. On the other hand, in some patients, such as those with RfH, a mineralocorticoid receptor antagonist does not play a determining role in the achievement of BP control, so new non-pharmacological strategies need to be tested in such cases. 

In addition to pharmacological treatment, the treatment of OSA with continuous positive airway pressure (CPAP) might be an alternative means of achieving BP control in RH subjects with OSA, due to its additional effect of lowering blood pressure in these patients [71,72,73,74]. Moreover, some RH or RfH patients might be particularly susceptible to a significant drop in BP with this treatment [75].

### 5.4. CPAP Treatment

**Figure 2:**
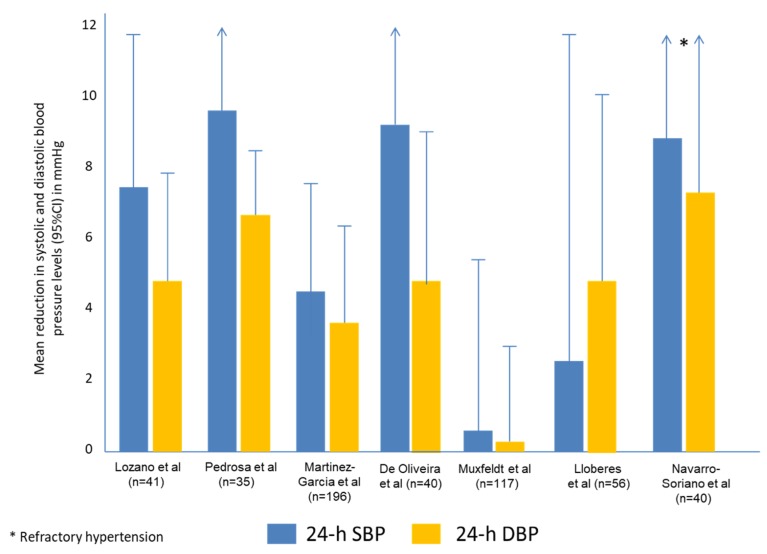
Effect of CPAP treatment on blood pressure in patients with resistant/refractory hypertension. Randomized controlled trials. SBP: systolic blood pressure; DBP: diastolic blood pressure.

Although the definitions of resistant and refractory hypertension have changed over time, the first study that can be considered an analysis of the effect of CPAP treatment on blood pressure levels in patients with resistant/refractory hypertension was published by Logan et al. in 2003 [76]. More specifically, the authors analysed the acute effect of CPAP on 11 RH patients. During one single night application, CPAP reduced systolic (S) BP by 12.3 mmHg and diastolic (D) BP by 4.8 mmHg. These reductions persisted after two months of treatment, especially during the night. The authors concluded that randomised clinical trials were needed to confirm their results. Since then, more than 20 studies have been published on different aspects of the effect of CPAP treatment in patients with RH or RfH: nine randomised controlled trials [RCT] [17,71,77,78,79,80,81,82,83], two of them on the effect on aldosterone concentration [79,82] and one on the effect on leptin concentration [84] (Table 2); four observational trials [84,85,86,87]; five meta-analyses and systematic reviews [72,88,89,90,91]; and, finally, five studies on additional topics, such as methodology [92], personalised medicine [75], adherence to CPAP treatment [93] and the combined effect of CPAP and renal denervation [63]. The first RCT on the effect of CPAP on BP levels in RH was performed by Lozano et al. in 2010 [77] in 64 patients with ABPM-confirmed RH. Patients treated with CPAP (*n* = 20) showed a decrease in 24 h diastolic BP (4.9 ± 6.4 vs. 0.1 ± 7.3 mmHg, *p* = 0.027) compared with the control group without CPAP. Moreover, the number of patients with a dipping pattern significantly increased in the CPAP group (51.7% vs. 24.1%, *P* = 0.008). Patients who used CPAP > 5.8 h showed a greater reduction in 24h BP (6.98 mmHg in diastolic blood pressure (DBP) and 9.71 mmHg in systolic blood pressure (SBP)). The largest RCT on the effect of CPAP on BP levels in RH patients was the HIPARCO study (NTC00616265) undertaken by the Spanish Sleep Network [17], in which 194 patients were randomised to CPAP (*n* = 98) or no CPAP (*n* = 96) over 3 months. The CPAP group achieved a greater decrease in 24 h mean BP (3.1 mm Hg [95% CI, 0.6 to 5.6]; *P* = 0.02) and 24 h DBP (3.2 mm Hg [95% CI, 1.0 to 5.4]; *P* = 0.005), but not in 24 h SBP (3.1 mm Hg [95% CI, 0.6 to 6.7]; *P* = 0.10), compared with the control group (Figure 2). Moreover, the percentage of patients with nocturnal BP dipper pattern after the 12 week follow-up was greater in the CPAP group (35.9% vs. 21.6%; adjusted odds ratio [OR], 2.4 [95% CI, 1.2 to 5.1]; *P* = 0.02). Finally, as seen in previous studies, there was a significant positive correlation between hours of CPAP use and the decrease in 24 h mean BP (*r* = 0.29, *P* = 0.006), SBP (*r* = 0.25; *P* = 0.02) and DBP (*r* = 0.30, *P* = 0.005.

The last published meta-analysis was performed by Lei et al. [88] and included six RCTs. The pooled estimates of the changes in SBP and DBP (as assessed by 24h-ABPM) were 5.40 mmHg (95% CI: 9.17 to 1.64; *p* = 0.001; and 3.86 mmHg (95% CI: 6.41 to 1.30; *p* = 0.00001), respectively. After this meta-analysis, an additional RCT was published by a French group (RHOOSAS study) [81] in 2018. Sixty-two patients with RH who were taking a mean of 3.7 antihypertensive drugs were included. Sixty percent of the patients presented OSA. Three months of effective CPAP significantly decreased night-time SBP by 6.4 mmHg and heart rate by 6.0 bpm, compared to sham CPAP. Moreover, their leptin concentrations were significantly lower those of non-OSA patients (9 (6; 15) vs. 17 (6; 29) ng/mL) but these increased after six months of CPAP.

Some interesting additional data on the effect of CPAP in patients with RH or RfH hypertension can be found in some other studies: Campos-Rodríguez et al. [93] reported that patients with RH had good adherence to CPAP after one year of follow-up (74.5%), demonstrating that a long-term RCT on these patients is feasible. Furthermore, a post hoc analysis of the HIPARCO study concluded that the effect of CPAP on RfH is even greater than that seen in patients with RH (without RfH). In this study, a total of 98 patients were randomised to CPAP (19 with RfH and 79 with RH) and 96 were randomised to usual care (21 with RfH and 75 with RH). BP readings dropped more markedly in those patients with RfH than in those with RH, in both 24 h SBP (9 vs. 1.6 mmHg, *p* = 0.021) and 24 h DBP (7.3 vs. 2.3 mmHg, *p* = 0.074), especially at night (11.3 vs. 3.8, *p* = 0.121 and 8.8 vs. 2.2, *p* = 0.054). The adjusted difference between groups was statistically significant in 24 h SBP levels (7.4 mm Hg, *p* = 0.021) [80]. Moreover, one of the factors associated with the progression over time from an RH to an RfH phenotype in those patients with OSA was non-adherence to CPAP treatment [94]. Recently, Sapiña et al. [92] published the methodology for SARAH (NCT03002558). This will be a multi-centre, prospective, observational cohort study in which a total of 1371 patients with RH have been enrolled, and they will be followed up once a year for five years. At their inclusion, all the subjects will undertake an ABPM and a sleep study. Socio-demographic, clinical and cardiovascular variables will be collected at baseline and follow-up, and OSA patients will be managed according to local standard practice. Based on the OSA diagnosis and its treatment, three cohorts of RH patients will be defined: non-OSA, treated OSA and non-treated OSA. This study will help elucidate the long-term impact of OSA treatments on blood pressure control and cardiovascular outcomes in patients with RH. Along the same lines, the final results of the long-term follow-up of the patients from the HIPARCO study (HIPARCO-2 study) will be published soon, throwing light on the effect of CPAP on cardiovascular events and BP after five years of follow-up. 

Beyond the known effect on the reduction in the sympathetic activity of CPAP, this treatment has also been shown to affect aldosterone concentration. De Souza et al. [82] demonstrated that only optimal CPAP treatment reduced aldosterone excretion in patients with uncontrolled RH, while the effect was borderline in an intention-to-treat analysis. Although these results are not definitive, they do suggest that CPAP treatment might improve cardiovascular outcomes by reducing aldosterone excess in RH patients with OSA. Furthermore, a previous study by Lloberes et al. [79] on the same topic observed that CPAP achieved a significant decrease in aldosterone concentration (26.1 ± 11.2 vs. 18.9 ± 10.1 ng/dL; *p* < 0.041) in patients with white-coat RH. After adjustment, a weak but significant association was found between cumulative time spent with SaO_2_ below 90% and baseline aldosterone concentration (*p* < 0.047), and between changes in aldosterone concentration and changes in office DBP (*p* < 0.020).

Moreover, Sanchez de la Torre et al. [75] observed three miRNAs obtained from responders and non-responders to CPAP treatment from the HIPARCO study, which provided a discriminatory predictive model for a favourable BP response to CPAP (area under the curve: 0.92; *p* = 0.01). CPAP treatment also significantly altered a total of 47 plasma miRNAs and decreased aldosterone-to-renin ratios in the responder group (*p* = 0.016) but not in the non-responder group. 

Finally, another study analysed the additional effect of CPAP on renal denervation. Linz et al. [63] published data taken from 1868 patients on the SYMPLICITY registry (NCT01534299): 205 had self-reported OSA, and the authors concluded that renal denervation resulted in significant BP reductions after 6 months in hypertensive patients with and without OSA, regardless of CPAP usage in the case of the OSA patients. 

In conclusion, and taking into account all these published findings on the effect of CPAP on BP levels and other characteristics in patients with RH or RfH, it seems that:

1. The reduction in BP levels is greater in RH patients (especially in those with a lack of BP control) than in normotensive or well-controlled hypertensive patients (approximately 2 mmHg)
2. The degree of reduction in BP levels is similar to those achieved by some antihypertensive drugs, especially in patients with RfH.
3. There is a positive and linear correlation between the hours of use of CPAP and the reduction in BP levels.
4. CPAP treatment produces an increase in the percentage of the normal nocturnal BP dipper pattern.
5. CPAP decreases both the sympathetic activity and the level of aldosterone concentration in both RfH and RH patients, and these could constitute some of the pathophysiological action mechanisms involved in this treatment.
6. The effect on RH of CPAP treatment combined with other pharmacological or non-pharmacological interventions such as renal denervation and antihypertensive drugs is still not known.

## 6. Future Challenges

The relationship between RH (especially RfH) and OSA has been detected recently and so many future challenges still await us, and they need to be tackled urgently for several reasons. On the one hand, there are few therapeutic options available for patients with RH/RfH, despite their ominous prognosis, while on the other hand, the prevalence of OSA among these patients is significantly high, and the treatment with CPAP has proved effective in reducing BP levels, being established as a new therapeutic way. To the date, beyond the magnitude of the effect of CPAP on BP in RH patients that might be even more important in RfH subjects, there is the need to set agreed criteria regarding how to fit in the multidimensional management of RH/RfH (Table 3) the potential role of the beneficial hemodynamic effects of this OSA treatment. Table 4 details some of the most important challenges associated with this relationship.

## 7. Conclusions

Patients with RH/RfH have a high cardiovascular risk and few therapeutic options. Recently, a close relationship between these phenotypes of hypertensive patients and OSA has been discovered. Both diseases seem to alter similar pathophysiological mechanisms. This has meant that in clinical studies, the prevalence of OSA in patients with RH/RfH is very high and that treatment with CPAP, which eliminates nocturnal respiratory events, is able to decrease BP levels in a clinically significant way. There are still many unanswered questions that pose future challenges such as the ideal combined treatment in these patients, or the role that long-term CPAP can play in the incidence of cardiovascular events. However, given the great cardiovascular risk of these patients and their scarce therapeutic options, ruling out the presence of an OSA is important since this is a potentially treatable disorder.

## Figures and Tables

**Table 1:** Studies that have investigated the association between resistant or refractory hypertension and sleep apnoea. Only those studies which have used a sleep test (either respiratory polygraphy or conventional polysomnography) have been included. Studies that assessed sleep apnoea risk based on screening questionnaires have not been included.

Studies	Patients (n)	Age (Years)	Type of BP Measure SBP/DBP (mmHg)	Type of Sleep Study (AHI Threshold to Define OSA)	OSA Prevalence/AHI
Logan 2001 [13]	41 patients with resistant HT (24 men, 17 women)	57.2 (1.6) Men 54.6 (1.8) Women 58.3 (3.0)	24 h ABPM SBP: 149.0 (2.6) in Men, 150.6 (3.7) in women DBP: 86.3 (2.0) in Men, 83.7 (1.9) in women	PSG (AHI ≥ 10)	82.9% (96% in men, 65% in women) Mean AHI: 32.2 (4.5) in men, 14.0 (3.1) in women
Martinez-Garcia 2006 [29]	49 pts with resistant HT (40.8% men)	68.1 (9.1)	24 h ABPM SBP: 152.5 (13) DBP: 89.2 (8.5)	RP (AHI ≥ 10)	AHI ≥ 10: 71.4% AHI ≥ 30: 40.8% Mean AHI: 26.2 (19.5)
Gonçalves 2007 [35]	63 pts with resistant HT (21 men, 42 women) and 63 pts with controlled HT (23 men, 40 women)	59 (7) in both the resistant and controlled HT groups	24 h ABPM SBP: 141 (17) in the resistant HT group vs. 121 (10) in the controlled HT group DBP: 84 (12) in the resistant HT group vs. 74 (7) in the controlled HT group	RP (AHI ≥ 10)	71% in the resistant HT group vs. 38% in the controlled HT group (*p* < 0.001) Men: 86% vs. 52% (*p* = 0.016) Women: 64% vs. 30% (*p* = 0.002)
Prat-Ubunama 2007 [34]	71 pts with resistant HT	56.0 (9.9)	Office BP measurement SBP: 155.8 (27) DBP: 88.3 (15)	PSG (AHI ≥ 5)	85% (90% in men, 77% in women) Mean AHI: 24.1 (24.7) (Men 20.8, Women 10.8)
Lloberes 2010 [33]	62 pts with resistant HT (67.3% men)	59 (10)	24 h ABPM SBP: 139.1 (1.6) DBP: 80.9 (1.2)	PSG (AHI ≥ 5)	AHI ≥ 5: 90.3% AHI ≥ 30: 70% Mean AHI: 47.8 (23.4)
Pedrosa 2011 [16]	125 pts with resistant HT (43% men)	52 (10)	24 h ABPM SBP: 176 (31) DBP: 107 (19)	PSG (AHI ≥ 15)	AHI ≥ 15: 64% AHI ≥ 30: 32% Median AHI: 18 (interquartile range, 10–40)
Florczak 2013 [32]	204 pts with resistant HT (123 men, 81 women)	48.4 (10.6)	24 h ABPM Daytime SBP: 145 (19), DBP: 90 (13) Nightime SBP: 132 (19), DBP: 79 (12)	PSG (AHI ≥ 5)	AHI ≥ 5: 72.1% AHI ≥ 30: 26.5%
Ruttanaumpawan 2009 [14]	42 pts with resistant HT and 22 pts with controlled HT, matched for age, sex and BMI	56.5 (1.6) in resistant HT group, 60.1 (1.8) in controlled HT group	24 h ABPM in the resistant HT group SBP: 149 (2) DBP: 85 (1)	PSG (AHI ≥ 10)	81% in the resistant HT group vs. 55% in the controlled HT group (*p* = 0.03) Mean AHI: 24.9 (3.2) in the resistant HT group vs. 16.5 (2.7) in the controlled HT group (*p* = 0.13)
Johnson 2019 [37]	664 black participants with HT (205 men), of whom 96 (14.5%) had resistant HT	64.9 (10.6)	Office BP measurement	RP (AHI ≥ 15)	25.7% of all HT patients. Patients with resistant HT were 1.92 times more likely (95%CI 1.15–3.20) to have OSA, compared to those with controlled HT
Abdel-Kader 2012 [38]	407 patients (229 men, 178 women), distributed in: 224 from general population without chronic kidney disease, 88 non-dialysis-dependent chronic kidney disease, and 95 with end-stage renal disease	60.0 (7.2) for the non-chronic kidney disease, 52.2 (14) for the non-dialysis-dependent chronic kidney disease, and 53.8 (14.9) for the end-stage renal disease group	Office BP measurement Resistant HT was present in 4.9% of patients in the non-chronic kidney disease, 35.2% of the non-dialysis-dependent chronic kidney disease, and 22.1% of the end-stage renal disease group	PSG (AHI ≥ 30)	Resistant HT was associated with severe OSA in participants with end-stage renal disease (adjusted OR 7.1, 95%CI 2.2–23.2), but not in the non-chronic kidney disease (adjusted OR 3.5, 95%CI 0.8–15.4) or the non-dialysis-dependent chronic kidney disease groups (adjusted OR 1.2, 95%CI 0.4–3.7)
Bhandari 2016 [36]	Retrospective cohort study of 470,386 individuals from a health insurance database	65 (11)	HT and Resistant HT were identified by ICD-9 specific diagnoses codes SBP: 139 (20) DBP: 75 (13)	Sleep apnoea was identified by ICD-9 specific diagnoses codes or by dispensation of positive pressure therapy	9.6% in the resistant HT group vs. 6.8 in the non-resistant HT group (*p* < 0.01). Sleep apnoea was significantly more common in the resistant HT group compared to the non-resistant HT group (adjusted OR 1.16, 95%CI 1.12–1.19)
Martinez-Garcia 2018 * [15]	229 pts with resistant HT (63% men). Of these, 42 (18.3%) had refractory HT	58.3 (9.6) for the resistant HT group and 58.4 (8.5) for the refractory HT group	24 h ABPM Resistant HT SBP: 141.6 (11.2) DBP: 82.2 (10) Refractory HT SBP: 152.4 (13.9) DBP: 85.6 (11.8)	RP (AHI ≥ 5)	AHI ≥ 5 Resistant HT: 89.3% Refractory HT: 100% (*p* = 0.027) AHI ≥ 30: Resistant HT: 48.6% Refractory HT: 64.3% (*p* = 0.044)

* This study investigate the association between OSA and refractory hypertension. HT: Hypertension; BMI: Body Mass Index; OSA: Obstructive Sleep Apnoea; SBP: Systolic Blood Pressure; DBP: Diastolic Blood Pressure; AHI: Apnoea–Hypopnoea Index; ABPM: 24h-Ambulatory Blood Pressure Monitoring; RP: Respiratory Polygraphy; PSG: Polysomnography; ICD: International Classification of the disease.

**Table 2:** Characteristics of randomised clinical trials on the effect of continuous positive airway pressure on blood pressure levels in patients with sleep apnoea and resistant/refractory hypertension.

Studies	Randomisation (Complete Follow-Up)	Age	BMI	ESS	Anti-HT Drugs	SBP/DBP (mmHg) at Entry	BP Measure	AHI, Sleep Study	CPAP Use	Follow-Up
Lozano et al., 2010 [77]	29 to CPAP 35 to control	59.2 (9.9)	30.8 (5)	6.14 (3.3)	3.48 (0.57)	129.9 (13.7)/76 (10)	ABPM	52.3 (21.5) Full PSG	5.6 (1.52)	3 months
Pedrosa et al., 2013 [83]	19 to CPAP 16 to control	56 (1)	32 (28–39)	10 (1)	4 (4–5)	162 (4)/97 (2)	ABPM	29 (24–48) PSG	6.01 (0.2)	6 months
Martinez-Garcia et al., 2013 [17]	98 to CPAP 96 to control	56 (9.5)	34.1 (5.4)	9.1 (3.7)	3.8 (0.9)	144.2 (12.5)/83 (10.5)	ABPM	40.4 (18.9) RP	5 (1.9)	3 months
De Oliveira et al., 2014 [78]	24 to CPAP 23 to sham	59.4 (7.7)	29.8 (4.4)	10 (6–15)	4 (1)	148 (17)/88 (13)	ABPM	20 (18–1) RP	5.3 (4.1–7.1) Median (IQR)	8 weeks
Muxfeldt et al., 2015 [71]	57 to CPAP 60 to control	60.5 (8.2)	33.4 (5.3)	11 (6)	5 (3–8)	129 (16)/75 812)	ABPM	41 (21) PSG	4.8 (median)	6 months
Navarro et al., 2019 * [80]	23 to CPAP 19 to control	61.1 (8.3) vs. 56.7 (9)	34.9 (5.4) vs. 34.1 (6.8)	9 (4) vs. 8.9 (3.8)	5 (5–6) vs. 5 (5–6) (median, IQR)	154.1 (12.2)/82.9 (14.2) 149(11.5)/84.1 (10.8)	ABPM	42.7 (17.2) vs. 40.4 (20.3) RP	5.2 (1.5)	3 months

* This study investigate the effect of CPAP on refractory hypertension. Data expressed by mean (standard deviation), unless indicated otherwise. BMI: Body Mass Index; ESS: Epworth Sleepiness Scale; anti-HT: antihypertensive; SBP: Systolic Blood Pressure; DBP: Diastolic Blood Pressure; AHI: Apnoea Hypopnoea Index; CPAP: Continuous Positive Airway Pressure; ABPM: 24h-Ambulatory Blood Pressure Monitoring; RP: Respiratory Polygraphy; PSG: Polysomnography; IQR: Interquartile range).

**Table 3:** The effect on BP and on OSA severity of different treatment strategies in subjects with resistant/refractory hypertension.

Treatment Strategy	Effect on BP	Effect on OSA
***Lifestyle and diet measures***		
Sodium restriction	Decrease of BP (*)	Improvement of OSA severity
Weight reduction	Decrease of BP (*)	Improvement of OSA severity
Regular physical exercise	Decrease of BP	(*)
Specific diet (Mediterranean, DASH)	Decrease of BP (*)	(*)
***Antihypertensive drugs***		
Diuretics	Decrease of BP	Improvement of OSA severity
Spironolactone	Decrease of BP (Fourth drug of choice)	Improvement of OSA severity
Other antihypertensive drugs	Decrease of BP	(*)
***Invasive procedures***		
Renal ablation	Probable decrease of BP (*)	Improvement of OSA severity
*OSA treatment (devices)*		
CPAP treatment	Decrease of BP especially in RfH	Control of the disease.
Other OSA treatments	Decrease of BP (*)	Control of the disease.

(*) Further research is needed.

**Table 4:** Future challenges in the relationship between RH/RfH and OSA.

To understand the pathophysiological mechanisms that distinguish RH and RfH, and how OSA and CPAP treatment can influence them.
To assess the best combined therapeutic strategy in patients with RH/RfH and OSA.
To determine the added value of CPAP to the different antihypertensive treatments, including renal denervation.
To analyse the effect of long-term CPAP on blood pressure and cardiovascular events in patients with RH/RfH.
To determine the various biomarker predictors of a good BP response to CPAP.
To group homogeneous clinical phenotypes in terms of clinical presentation, prognosis and response to treatment.
To determine the role of confounders in the relationship between RH/RfH and OSA, particularly obesity.
The contrast in office BP measures and 24 h ABPM results makes it possible to define different phenotypes of RH, according to whether the monitoring results are congruent (controlled or sustained) or not (white coat or masked). At present, the predictive value of each phenotype with respect to the effect of CPAP is still unknown.
